# Osteochondritis dissecans and Osgood Schlatter disease in a family with Stickler syndrome

**DOI:** 10.1186/1546-0096-7-4

**Published:** 2009-02-04

**Authors:** Ali Al Kaissi, Klaus Klaushofer, Franz Grill

**Affiliations:** 1 Ludwig-Boltzmann Institute of Osteology at the Hanusch Hospital of WGKK and AUVA Trauma Centre Meidling, 4th Medical Department, Hanusch Hospital, Vienna, Austria; 2 Orthopaedic Hospital of Speising, Paediatric Department, Vienna, Austria

## Abstract

**Purpose:**

Stickler syndrome is among the most common autosomal dominant connective tissue disorders but is often unrecognised and therefore not diagnosed by clinicians. Despite much speculation, the cause of osteochondrosis in general and osteochondritis dissecans (OCD) and Osgood Schlatter syndrome (OSS) in particular remain unclear. Etiological understanding is essential. We describe a pair of family subjects presented with OCD and OSS as a symptom complex rather than a diagnosis.

**Methods:**

Detailed clinical and radiographic examinations were undertaken with emphasis on the role of MRI imaging. Magnetic resonance imaging may allow early prediction of articular lesion healing potential in patients with Stickler syndrome.

**Results:**

The phenotype of Stickler syndrome can be diverse and therefore misleading. The expectation that the full clinical criteria of any given genetic disorder such as Stickler syndrome will always be present can easily lead to an underestimation of these serious inheritable disorders. We report here two family subjects, a male proband and his aunt (paternal sister), both presented with the major features of Stickler syndrome. Tall stature with marfanoid habitus, astigmatism/congenital vitreous abnormality and submucus cleft palate/cleft uvula, and enlarged painful joints with early onset osteoarthritis. Osteochondritis dissecans (OCD) and Osgood Schlatter syndrome (OSS) were the predominating joint abnormalities.

**Conclusion:**

We observed that the nature of the articular and physeal abnormalities was consistent with a localised manifestation of a more generalised epiphyseal dysplasia affecting the weight-bearing joints. In these two patients, OCD and OSS appeared to be the predominant pathologic musculoskeletal consequences of an underlying Stickler's syndrome. It is empirical to consider generalised epiphyseal dysplasia as a major underlying causation that might drastically affect the weight-bearing joints.

## Background

The osteochondrodysplasias are a clinically and genetically heterogeneous group of diseases that affect the development of the skeleton. Stickler syndrome is an autosomal dominant arthro-ophthalmopathy disorder linked to mutations in types II and XI collagen. Stickler syndrome, however, can manifest a wide range of variable and confusing phenotypic features, vary from dwarfism/marfanoid habitus to phenotypically normal individuals [1,2]. The disorder is characterised by enlarged joints, epiphyseal changes, mild platyspondyly and severe myopia with retinal detachment leading to blindness [3]. There may be cleft palate and hearing defects. Inheritance is autosomal dominant. The patient is usually tall and slender with peculiar facies and mild ligamentous laxity. The latter only manifested in early life period, which seldom later on turns to generalised ligamentous stiffness. The disorder is different than Marfan syndrome [4].

Osteoarthritis, scoliosis, spondylolisthesis, and Scheuermann's kyphosis are considered the paramount orthopedic abnormalities manifested by Stickler syndrome [1,2,5,6]. "Osteochondritis dissecans" (OCD), and "Osgood Schlatter syndrome" (OSS) are major enigmatic orthopedic conditions. Several hypotheses have emerged regarding the aetiology of osteochondritis dissecans [7-13]. Most of these hypotheses require prompt delineation to eliminate the speculative and to approach to definite corroborating evidences. Trauma has been regarded as the potential trigger in the development of OCD and OSS [14]. Osteochondritis dissecans (OCD), osteochondritis deformans (Perthes disease) and tibial tubercle avulsion fracture were described as part of the symptom complex in some heritable bone disorders [15-20]. Familial type of osteochondritis dissecans has been reported in the literature and autosomal dominant pattern of inheritance was suggested [21,22]. To the best of our knowledge this is the first clinical report describe the simultaneous occurrence of osteochondritis dissecans and Osgood Schlatter syndrome in connection with Stickler syndrome in two family subjects.

## Case presentation

### Patient 1

Patient 1 was referred to the orthopaedic department at the age of 15 years because of irritable bilateral knee pain since early childhood. Recently a 5-month history of left groin pain without any history of trauma was an additional complaint. At birth he was noted to have a submucus cleft palate. The submucus cleft palate was operated on at the age of 3 years. At preschool age, conductive hearing loss and vision errors of astigmatism were identified.

**Figure 1:**
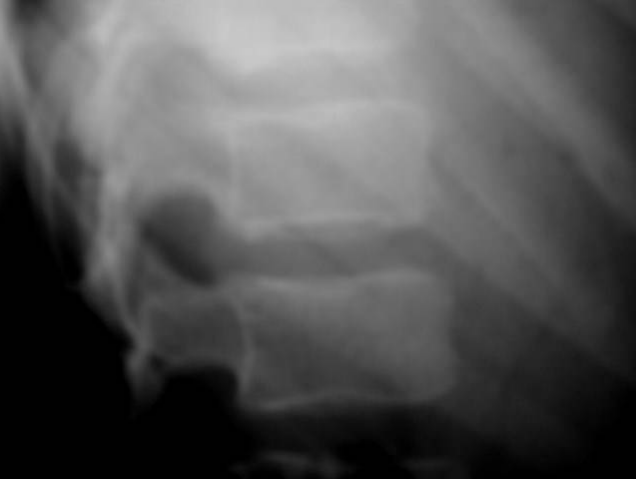
**Patient 1: Vertebral column lateral radiogram at age of 15 years showed platyspondyly with anterior end-plate irregularity associated with irregular surfaces of the superior and inferior apophyses**.

**Figure 2:**
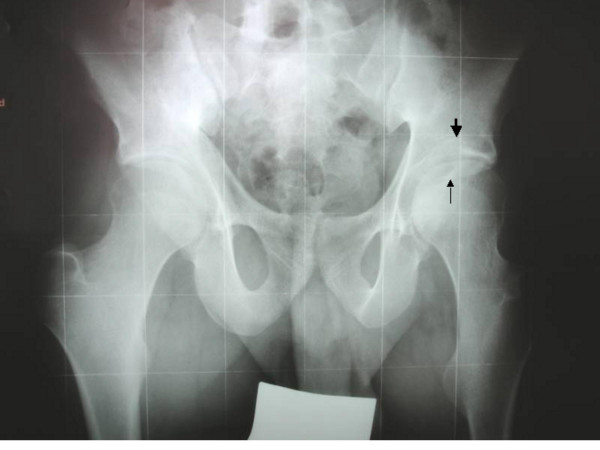
**Patient 1: Pelvis Anteroposterior radiograph at age of 15 years showed coxa valga, relative flattening of capital femoral epiphyses, joint space narrowing and irregularity, with features of left acetabular osteochondritis associated with necrotic adjacent femoral epiphysis (arrows)**.

**Figure 3:**
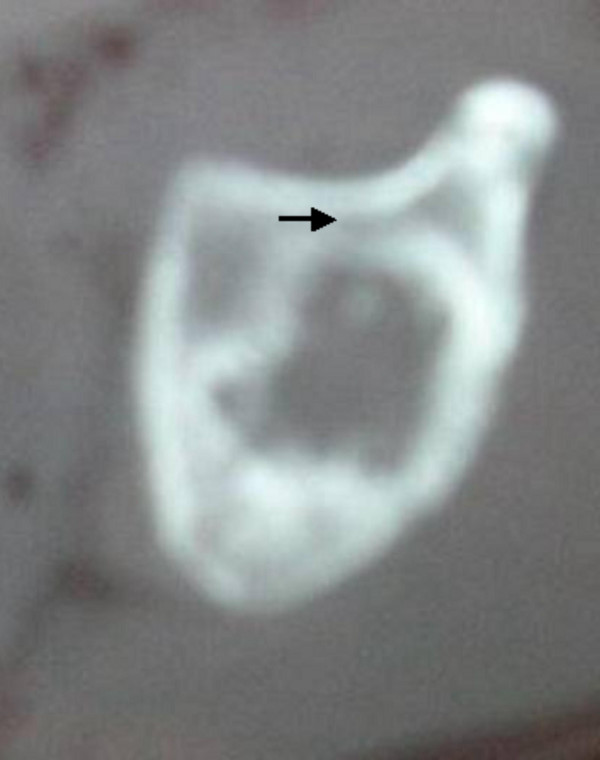
**Patient 1: Axial CT scan at age of 17 years shows a necrotic acetabular roof (arrow)**.

His subsequent course of development, particularly his motor development has shown significant retardation. Problems of getting off the floor and normal walking were bothersome. In his early life ligamentous hyperlaxity was a prominent hallmark. Neuromuscular disorders were ruled out. At school age, bouts of bilateral knee pain were experienced. Exercise intolerance became a major burden. Clinical examination showed a patient of tall stature, 187 cm (above 97^th^. th percentile). Minor dysmorphic features of flat nasal bridge, maxillary hypoplasia, high arched palate, and a small chin were present. Thoracic scoliosis associated with spine stiffness (figure 1). The left hip showed moderately restricted range of movements in abduction, adduction, and external rotation associated with limb enquality of 3 cm (figure 2, 3). An antalgic gait was observed with maximal tenderness over the anteromedial aspect of each knee. Relative quadriceps muscle atrophy with point tenderness was observed.

**Figure 4:**
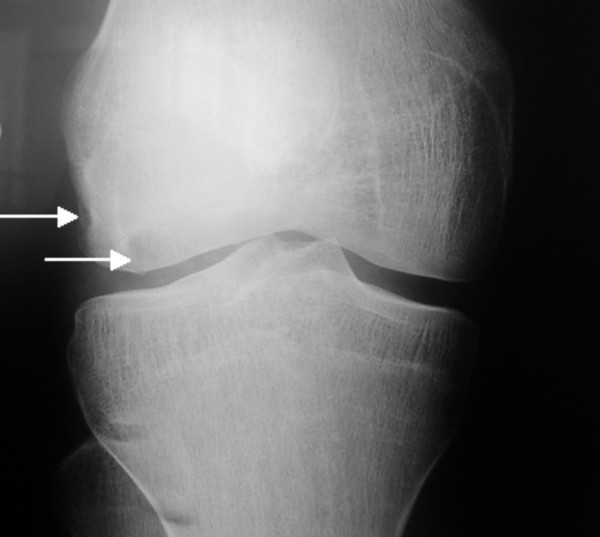
**Patient 1: Anteroposterior knee radiograph at age of 15 years shows osteochondritis dissecans of the distal femoral epiphysis with areas of evident epiphyseal necrosis (arrows)**.

**Figure 5:**
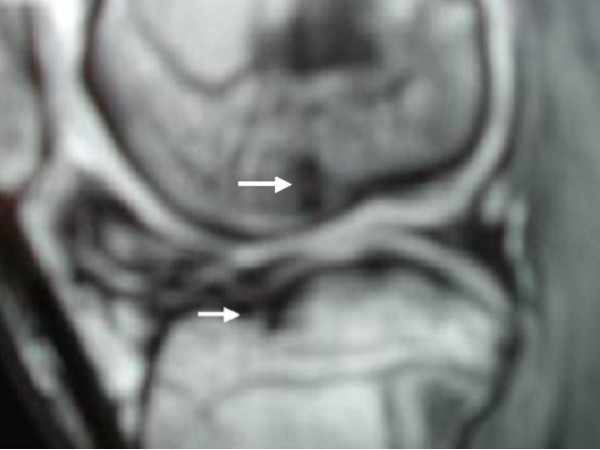
**Patient 1: Coronal MR image of the knee at age of 17 years shows progressive and lesion from the underlying subchondral bone with apparent anterior separation of the articular surface with evidence of epiphyseal friability**. Note the bony fragment had resulted in a concave crater on the femoral condyle with steeply sloping edges (arrows)

**Figure 6:**
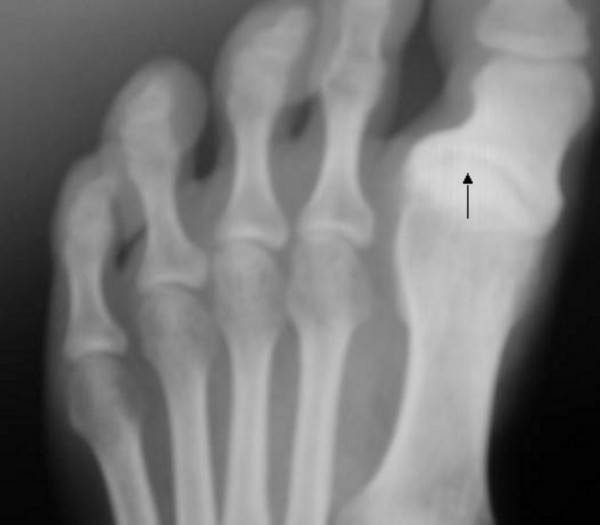
**Patient 1: Anteroposterior at age of 17 years foot radiograph showed progressive hallux varus with marked osteoarthritis of the base of the first toe**.

Wilson sign was positive (Wilson's sign is achieved through reproducing pain by internally rotating the patient's tibia during knee extension between 90° and 30° of flexion and then relieving that pain by externally rotating the tibia), in addition crepitus and pain with motion were notable as well. There was bilateral involvement of the knee, the lesions, however were typically asymmetrical in terms of size and symptoms (figure 4, 5). Bilateral hallux varus was associated with pain of maximal intensity over the base of the big toe (figure 6). Vigorous investigations of electromyography (EMG), electroencephalography (EEG) and Echo-cardio Doppler were normal. Complete blood count, and synovial fluid analysis (derived from the knee) ruled out septic arthritis. His ESR was unremarkable and he showed a negative rheumatoid factor. Other laboratory tests included serum lactate, serum pyruvate, creatine phosphokinase, and chromosomal study. The results of these tests were normal. His younger male sib has been described to have a similar tall stature and marfanoid habitus; he developed congenital vitreous abnormality in his early life. Unfortunately, the parents were unwilling to give us the access to examine this child.

### Radiographic examination of Patient 1 (figures 1, 2, 3, 4, 5, 6)

Orthopaedic treatment was primarily planned upon non-surgical intervention. These measures were initially successful, but later on pain recurs. It is highly likely that we might proceed with a mosaic arthroplasty, as a treatment of choice.

### Patient 2

Patient 2 is a 49-year-old-woman the aunt of patient 1, manifested a marfanoid habitus, with a height of 178 cm (above 97^th^. percentile). Craniofacially she exhibited prominent eyes (proptosis), epicanthal folds, small nose, flat nasal bridge, long philtrum, cleft uvula, and micrognathia. No history of hearing loss, but early childhood astigmatism was the main ophthalmological problem. Musculoskeletal examination showed relative generalised articular stiffness with features of multiple joint pain. Thoracic spine kyphosis in connection with Scheuermann's adolescent kyphosis (osteochondritis of the upper and lower cartilaginous vertebral end plates) associated with marked stiffness.

**Figure 7:**
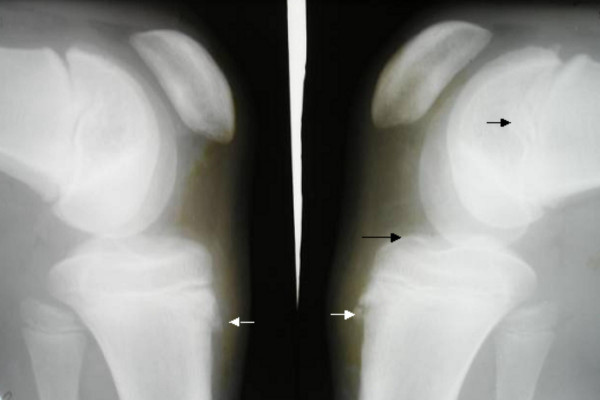
**Patient 2: Lateral radiograph of the knee with the leg internally rotated (10–20°) showed bilateral irregularity of the apophysis with separation from the tibial tuberosity**.

She developed pain in her knees at the age of 12 years. Pain worsened after sporting activities, which involved jumping and or running. Osgood-Schlatter syndrome was recognised. The patient was advised to restrict her activities. Oral anti-inflammatory medication, knee padding, and physical therapy associated with special exercises to improve the flexibility of the surrounding muscles were recommended. Symptoms, however, continued unabated into adulthood. At the age of 16 years prominent tibial tuberosity were apparent in the lateral view of the knees (fig 7). Tibial sequestrectomy associated with insertion of bone pegs was performed successfully at the age of 20 years. Weight-bearing joint pain over the hips, knees and ankle is still a dilemma for this patient. Nevertheless, weakness of the quadriceps muscles persisted after the operation.

## Discussion

Stickler syndrome is thought to be one of the most common autosomal dominant connective tissue disorders [1,2]. As pointed out by Opitz [23], this syndrome can be extremely variable. At birth the only features may be those of Pierre Robin association (cleft palate, micrognathia and glossoptosis). Van den Elzen et al [24] found that 15% of a series of children with Pierre Robin association had Stickler syndrome. Radiological examination at this time may reveal coronal clefts of the vertebrae, mild platyspondyly and flaring of the metaphyses of the long bones (features of the Weissenbacher-Zweymuller syndrome) [25]. As the child grows older these features become normal, although a mild epiphyseal dysplasia may develop. Lewkonia [26] described the orthopaedic manifestations in 53 patients with Stickler syndrome. 34% had scoliosis, 74% endplate vertebral anomalies, 43% had platyspondyly and 64% had Schmorl nodes. He suggested that total joint arthroplasty is also common and may be needed before the age of 20 years.

Harkey et al [27] reported a case with disc herniations causing paraplegia. Rose et al., [28] studied 53 patients in 24 families. 34% of patients had scoliosis, 74% vertebral endplate abnormalities, 64% Schmorl's nodes, 43% platyspondyly and 43% Scheuremann-like kyphosis. 85% of adults reported chronic back pain. Variants of Stickler syndrome have been designated STL1 (vitreous type with mutations in COL2A1 gene), STL2 (early onset hearing loss and mutations in the COL 11A1 gene) and STL3 (non-ocular type caused by mutations in the COL11A2 gene). Numerous genetic mutations of Stickler syndrome cases are STL1 variety and are caused by mutations in the COL2A1 gene [29]. In practice, however, there are difficulties in obtaining molecular analysis because of the size, complexity and the number of gene involved and the high cost of these investigations.

Early degenerative arthritis or osteoarthritis with radiographic evidence of bone destruction might occur in patients with osteochondrodysplasias [30]. The pathological mechanism of OCD that manifests as a pathological spectrum including softening of the overlying articular cartilage with an intact articular surface, early articular cartilage separation, partial detachment of an articular lesion, and osteochondral separation with loose bodies. In most instances, however, the definitive causation is unclear and remains controversial [7-11]. Fairbank supports the theory of trauma [14]. Mubarek and Carroll described a family in which 12 out of 31 members in four generations had proven osteochondritis dissecans of the medial femoral condyle and 8 a probable lesion with autosomal dominant inheritance [21]. Kozlowski and Middleton described a familial, rather than a syndromic form of osteochondritis dissecans [22].

Murray et al [31] reviewed 32 knees in 26 patients who had previously undergone arthroscopic debridement for symptomatic osteochondritis dissecans (OCD) of the knee. The approximate incidence of osteochondritis dissecans (OCD) is 3 to 6/10,000 in adults. Much of the early literature collected osteochondritis dissecans, osteochondral fracture, and accessory ossification in one basket! Franceschi et al [32] reported the first case of simultaneous location of ostechondroses of the two ossification centers of both patellae in a 9-year-old boy. The boy was considered as an atypical case of osteochondrosis because he presented with bilateral knee osteochondroses, involving simultaneously the primary ossification centre (Kohler syndrome) and the secondary ossification centre (Larsen syndrome) of the patella. Despite the bilateral involvement, no comprehensive clinico-radiographic documentation was made to further delineate the disorder.

Hoornaert et al [33] observed (OCD) in three families who manifested features of R519C COL2A1 genotype. They considered these mutations are of diagnostic relevance in which some of these mutations seem to cause unusual phenotypes such as Stickler syndrome. Liberfarb et al [34] evaluated a cohort of clinically diagnosed Stickler syndrome for which the causative mutation has been identified. They concluded that the molecular determination of a mutation could predict the occurrence of Stickler syndrome, but it was difficult to predict the severity of the phenotype on the basis of genotype.

Osgood Schlatter syndrome (OSS), or tibial osteochondrosis, is the most common traction apophysitis and overuse injury in the knee of adolescent athletes. The condition is caused by multiple submaximal avulsion fractures of the patellar tendon attachment to the tibial tubercle, when the tibial tubercle is in the apophyseal stage and the secondary ossification center has appeared. The aetiology of (OSS) also remains controversial [12,13,16,17,35].

## Conclusion

In their early observations Stickler and co-workers described the adverse implications for the development of loose osteocartilaginous bodies that cause joint locking in patients with ophthalmoarthropathy. Finally we wish to stress that the great diversity and the severity of ophthalmological/articular pathologic conditions is a fundamental reason behind the underestimation of this serious disorder.

## Competing interests

The authors declare that they have no competing interests.

## Authors' contributions

AAK: drafted the manuscript and analysed the data. KKH participated in the design of the study. FG conceived of the study, and participated in its design and coordination. All authors read and approved the final manuscript.

## Consent

Written informed consent was obtained from the patient for publication of this case report and accompanying images. A copy of the written consent is available for review by the Editor-in-Chief of this journal
